# Comparison of the AO/OTA 1996/2007 and 2018 pelvic ring fracture classifications

**DOI:** 10.1007/s00402-024-05557-2

**Published:** 2024-10-09

**Authors:** Jan Lindahl, Axel Gänsslen, Jan Erik Madsen, Dietmar Krappinger

**Affiliations:** 1 Department of Orthopaedics and Traumatology, Helsinki University Hospital, and University of Helsinki, Haartmaninkatu 4, 00290 Helsinki, Finland; 2 Trauma Department, Hannover Medical School, Hannover, Germany; 3 Department of Trauma and Orthopedics, Johannes Wesling Hospital, Minden, Germany; 4 Division of Orthopaedic Surgery, Oslo University Hospital, Oslo, Norway; 5 Institute of Clinical Medicine, University of Oslo, Oslo, Norway; 6 Department of Orthopaedics and Traumatology, Medical University Innsbruck, Innsbruck, Austria

**Keywords:** Pelvic ring injury, Tile classification, AO classification, Young–Burgess classification, AO/OTA classification 2018

## Abstract

Pelvic ring fractures may present with relevant mechanical and haemodynamic instability. Classifications of the bony or ligamentous injuries of the pelvic ring are well established. The most common classifications used analyse the injury mechanisms and the resulting instability of the pelvic ring structure. Fracture classifications should be simple and easy to use, comprehensive, and radiographically and anatomically based, resulting in a hierarchical alphanumeric order of types and subtypes and thereby allow adequate treatment decisions based on a high degree of inter- and intraobserver reliability. In 2018 a new AO/OTA pelvic ring fracture and dislocation classification was published that combined the most commonly used “historical” classification schemes, e.g. the Tile/AO classification and the classification according to Young and Burgess. Compared with these older classifications, several relevant changes were integrated in the 2018 edition. The changes between the AO/OTA 1996/2007 and 2018 classifications were analysed in detail. Overall, several problems were identified regarding the type-B pelvic ring injury classification. These changes may result in difficulties in classifying pelvic ring injuries and thereby prevent relevant comparisons between former and future clinical studies on pelvic injuries.

Level of Evidence: V.

## Introduction

Pelvic ring injuries can be simple, when only decisions on treatment type of the fracture regions must be considered. In contrast, treatment of pelvic injuries as part of multiply injured patients should additionally focus on the accompanying injuries and concomitant haemodynamic status of the patient.

Classifications of the bony injury represent the basis of understanding the pelvic injury. The main aim using any classification is to estimate the instability of the pelvis and thereby assess the risk of pelvic bleeding.

Malgaigne first recognized the ring structure of the pelvis in 1847 and distinguished between single and double ring disruptions [18]. Most of  the following classifications were oriented on the anatomic fracture morphology with the difficulty of the high variability of different pelvic fracture locations.

In 1961, Pennal and Sutherland correlated the suspected accident mechanisms with the resulting fracture types and identified the following three force vectors leading to reproducible pelvic ring lesions: anterior–posterior force direction, lateral compression and vertical shear (VS) injury [27]. The main problem using these mechanisms alone was, that the aspect of stability or instability, essential for clinical decision making, was not analysed in detail. This led to a further subdivision of each instability type into three degrees of instability within the anterior–posterior compression (APC) and lateral compression (LC) group of injuries by Young and Burgess [6].

The combination of the injury mechanism and the degree of instability represents a non-linear classification. Marvin Tile modified the original Pennal classification [28] and integrated his thoughts into the hierarchical AO classification scheme [38, 39].

The aim of this study was to analyse the value of the Tile and Young-Burgess classifications and explore the changes made into the new AO/OTA fracture and dislocation classification compendium published in 2018 [16] and to compare these to the previous AO/OTA classifications [21, 24].

## Aims of classifications

Based on the historical work of Maurice Müller [23] and others, a classification should:

- Be simple and easy to use [17]
- Include the possibility of logical treatment decisions [11, 37, 39]
- Be useful and thus help the surgeon select an appropriate treatment concept for each conceivable fracture and allow a reasonable estimation of the prognosis based on the chosen therapy [23]
- Be as homogeneous as possible and thus create clearly separated patient groups [39]
- Enable comparison with other working groups and an estimation of prognosis and treatment [2]
- Be functional, with a high degree of inter- and intraobserver reliability [23]

### Osteoligamentous pelvic ring classifications

The classifications predominantly used for pelvic ring injuries in daily practice in the last three decades have been the Tile or AO/OTA classification [26] and the Young/Burgess classification [42].

The Young-Burgess classification, which is based on the main force vector acting on the pelvis is frequently used in North America. The Tile classification, which is based on the amount of pelvic instability, and the AO/OTA classification, which is based on the Tile classification, are predominantly used in Europe.

In 2018, the AO/OTA pelvic ring fracture classification was modified to a newer version, with an attempt to integrate both the AO and the Young-Burgess classifications [16].

### Tile classification

The Tile classification is based on the main injury mechanisms acting on the pelvis (APC, LC, and VS) and was reported early in the 1980s [28, 41]. Tile developed his instability-based classification [38] focused on the involvement and integrity of the posterior pelvic ring structures. This classification was integrated into the hierarchical AO concept of three main fracture or injury types with increasing mechanical instability of the pelvic ring :

- Type A: stable, minimally displaced
- Type B: rotationally unstable, vertically stable
- Type C: rotationally and vertically unstable

**Table 1:** AO/OTA classification of pelvic ring injuries according to Tile’s recommendation [24, 34]

Type A: Stable pelvic ring
A1: Avulsion of the innominate bone
A2: Stable iliac wing fracture or stable minimally displaced ring fractures
A3: Transverse fractures of the sacrum and coccyx below the SI-joint level
Type B: Partially stable pelvic ring
B1: Open-book injury
B2: Lateral compression injury
B2.1: Ipsilateral type
B2.2: Contralateral type (bucket handle)
B3: Bilateral B injuries
Type C: Complete unstable pelvic ring
C1: Unilateral
C1.1: Ilium
C1.2: Sacroiliac dislocation or fracture dislocation
C1.3: Sacrum
C2: Bilateral, one side B, one side C
C3: Bilateral C lesions

With increasing understanding of the injury mechanism and the stability-based concept of the pelvic ring, this primary classification scheme was changed [34, 39, 40], resulting in the comprehensive AO and OTA classification in 1996 (Table 1) [23, 24, 26, 28, 30].

The Tile classification is of prognostic relevance regarding mortality, functional results, and neurological long-term sequelae [10, 29].

An increase in mortality rate was observed from stable to completely unstable injuries: 8.8% for type A- injuries, 13.8% for type B-injuries, and 25% for type C-injuries [12]. Within the type-B group, an increase of mortality was seen from B1 to B3 injuries [25]. The mortality rate after B3 injuries (bilateral posterior type B lesions) was comparable to the overall group of type-C injuries; as in severe open book injuries (B3), a high rate of concomitant intrapelvic vessel injuries can be expected. Type-C2 injuries were associated with the highest mortality [3]. Rommens et al. reported a three-fold higher mortality rate in type-C injuries [31].

The instability of the pelvic ring was associated with reduced reconstruction capabilities. Anatomic reductions decreased from 93.5% in type-B1 to 75% in type-B2/B3 and to 62.7% in type-C injuries with a corresponding poorer functional outcome [31]. B1 injuries were associated with less favourable functional results and higher rates of neurological and urological lesions than type-B2 or -B3 injuries [31, 32].

### Young–Burgess classification

This classification analyses the degree of different injury force vectors. Radiographic analysis identified the following three main force vectors [42]:

- APC (external rotation of the hemipelvis)
- LC(internal rotation of the hemipelvis)
- VS

For APC injuries symphysial separation < 2.5 cm was not combined with SI-joint instability [42].

 injuries are most often a combination of characteristic horizontal (comminuted) pubic rami fractures and sacrum fractures [6, 42].

VS injuries result from severe vertical force vectors (fall from height), involving one or both sides of the posterior pelvis [15]. Severely displaced VS injuries, especially with caudal hemipelvic displacement, are associated with significant arterial injury, even requiring hemipelvectomy in select cases [19]. In cases with at least two of these suspected force vectors, the injury was termed complex fracture pattern (CM).

Young and Resnik summarized these radiographic findings (Table 2) [42], and several studies have evaluated the clinical relevance of the Young-Burgess classification and its subtypes.

**Table 2:** Pelvic ring fracture classification according to Young and Burgess with region-specific parameters [42]

Fracture type	Pubic rami fractures	Symphysis pubis diastasis	Sacral fractures	SI-joint diastasis	Iliac wing fractures	Hemipelvic displacement	Acetabular fractures
LC 1	Horizontal	No	Ipsilateral	No	Rare	No	Medial wall
LC 2	Horizontal	No	Lateral	No	Oblique	Minimal medial	Medial wall
LC 3	Horizontal	No	Lateral	Contralateral	Oblique/crush	Medio-lateral*	Medial wall
APC 1	Vertical	< 2.5 cm	No	No	No	No	AC+/-PC**
APC 2	Vertical	> 2.5 cm	No	Anterior	No	Anterior lateral	AC+/-PC**
APC 3	Vertical	Variable	Rare***	Complete	No	Lateral	AC+/-PC**
VS	Vertical	Variable****	Vertical	Variable****	Variable****	Vertical	Roof

*Ipsilateral medial, contralateral lateral

**AC=Anterior column; PC=posterior column

***Sacral avulsion fracture of the pelvic floor ligaments

****Vertical displacement

Compared to LC-injuries, APC-injuries are more frequently associated with pelvic vascular injury, retroperitoneal haematoma formation, primary haemodynamic shock, and 24-h transfusion needs due to stretching of the vascular structures. After LC-injuries, pelvic vascular injury is supposed to be a result of direct fracture fragment contact [8]. The rate of haemodynamic instability and additional intra-abdominal injuries increases from APC1 to APC3 [8].

APC injuries are graded into three subtypes, depending on the amount of symphyseal separation and posterior instability. As the pelvic antero-posterior (AP) x-ray is only a static radiograph, underestimation of the initial displacement is common [13], demonstrating occult instabilities [33].

Starr et al. reported the highest mortality rates in LC3 and APC3 injuries [36]. Recently, LC3, APC2, and APC3 injuries were shown to be associated with higher transfusion requirements than LC1, APC1, and VS injuries; no association with additional injuries of the head, chest, or abdomen was observed [20].

When comparing mechanically more stable (LC1, APC1) and unstable (LC2, LC3, APC2, APC3, VS, CM) fracture patterns, unstable fractures better predicted mortality rates, concomitant abdominal injury rates, and transfusion requirements [20].

Overall, the Young-Burgess classification is useful in estimating haemodynamic instability. The main disadvantage of the Young and Burgess classification is its limited value regarding guidance of osteoligamentous treatment [1].

### AO/OTA classification 2018

The existing AO/OTA classification [21,23, 24,26] was modified in 2018 [16] with integration of parts of the “old” AO/OTA classification and the Young-Burgess classification [16].

Type-A injuries are in accordance with the AO/OTA 1996 and 2007 pelvic ring fracture classifications.

The main changes in the 2018 classification are in the type B-injuries. It is confusing that the B1 and B2 classes have swapped numbers from the previous classifications. The classic B1 and B2 have been removed. Type B-injuries are still defined as incomplete disruptions of the posterior arch of the pelvic ring structure. This fracture type is divided into three groups and eight subgroups (Table 3).

**Table 3:** Analysis of the 2018 AO/OTA type-B pelvic ring injury group addressing the changes of injury mechanisms, instability patterns, and change of hierarchy [16,24, 38]

B1: Incomplete disruption of posterior arch, no rotational instability:
B1.1: Lateral compression fracture (LC1) = Tile B2
B1.2: Open book fracture (APC1) = Tile B1
B2: Incomplete disruption of posterior arch, rotationally unstable, unilateral posterior injury
B2.1: Lateral sacral compression fracture + internal rotation instability (LC1) = Tile B2
B2.2: Lateral compression fracture of the ilium (crescent type) with internal rotation instability (LC2) = Tile C?
B2.3: Open book or external rotation instability (APC2) = Tile B1
B3: Incomplete disruption of posterior arch, rotationally unstable, bilateral posterior injury
B3.1: Internal rotation instability on one side and external rotation instability on the contralateral side (LC3) = Tile B3
B3.2: Bilateral lateral compression (LC) sacral fracture (LC1) = Tile B3
B3.3: Bilateral open book (APC2) = Tile B3

Interestingly, several modifiers of morphological anterior ring injuries were integrated, which were identical for type-B1 and -B3 injuries, while only a pure symphyseal disruption was added for type-B2 injuries.

Type-C injuries are defined as a complete disruption of the posterior pelvic ring and are in accordance with the previous AO/OTA classification. However, transverse sacral fracture at the S1-S2 level with vertical sacral fracture components forming U-, Y- (lambda), or H-shaped sacral fractures are not included in these fracture classifications.

Additionally, modifiers of different anterior and posterior fracture morphologies were integrated, but inconsistent. For example, sacroiliac joint fracture dislocations were added for C1 injuries, while these injury types were missing in type-C2 and -C3 injuries.

## Discussion

It seems useful to combine the most commonly used osteoligamentous pelvic ring injury classifications schemes (AO/OTA and Young-Burgess) with the potential aim of acquiring information on mechanical and haemodynamic instability. Nevertheless, the 2018 version of the AO/OTA classification has several of confusing issues.

A classification should be simple and easy to use [17], providing the possibility of logical treatment decisions [11, 37, 40] and thus helping the surgeon choose a fracture type-based treatment concept. Additionally, a prognostic estimation should be possible for the selected treatment[23].

The present universal and comprehensive 2018 fracture classification still favours prognostic relevance with increasing pelvic ring instability from A1 to C3 injuries. However, type B-injuries have become unclear. In contrast to prior versions, the B1 injury is no longer a rotationally unstable injury. These injuries are now defined as incomplete disruptions of the posterior arch without rotational/posterior instability and are further subdivided into the two subgroups B1.1 and B1.2, which are LC1 and APC1 injuries according to the Young-Burgess classification.

An isolated anterior fracture of the pelvic ring is uncommon [39]. When such a fracture is detected, a posterior disruption or some sign of compression should be sought. The basic rule is that when the pelvic ring is disrupted in one place, there is an injury in another portion of the ring [38]. A radioisotope bone scanning study by Gertzbein and Chenoweth [14] showed that undisplaced fractures of the pubic rami were invariably accompanied by a second injury located in the sacroiliac area. This second injury might be a fracture, a torn ligament, or a disrupted ligament attachment. In a study of post-mortem material, Bucholz [5] confirmed the presence of a posterior lesion in all cases. Exceptions to this rule are avulsion fractures of the iliac spines and isolated iliac wing fractures.

B2 and B3 injuries now exclusively represent rotationally unstable injuries with partially persistent posterior stability. The B2.1, B2.3, and all B3 injuries now represent the classical type-B injuries according to Tile and the former AO classification, which most often only require anterior pelvic ring fixation, while the B2.2 injuries are primarily focused on the injury mechanism (LC), resulting in a crescent type fracture.

Crescent fractures are morphologically fracture dislocations of the SI-joint and therefore represent unstable posterior pelvic ring injuries, with anterior and posterior fixation requirements. this has been a longstanding problem with the Young-Burgess classification, as the morphology of the crescent fracture represents a complete posterior disruption in the Tile classification. Thus, there is still a significant “grey zone” regarding analysis of instability and treatment concepts.

Another disadvantage of the present 2018 fracture classification is the incomplete description of the posterior ring involvement. In C1 injuries, an injury to the SI-joint can be modified by description of a sacroiliac joint fracture dislocation, while in C2 and C3 injuries, this option does not exist.

This implies confusion when classifying these injuries, as with these modifications the overall classification scheme is not simple and easy to use [17] and does not provide logical treatment decisions [11, 37, 40] or help the surgeon choose a fracture type-based treatment concept.

The 1996 AO/OTA classifications described the B1 injury with all its subtypes as an open book injury, while B2 injuries were classically LC injuries. With the new 2018 classification, the B1 type is a mix of open book (B1.2) and LC injuries (B1.1) and therefore comparisons to former treatment specific analyses are no longer possible.

An unsolved problem with every classification is the crescent fracture injury. A crescent fracture is a combination of a ligamentous disruption of the inferior portion of the sacroiliac joint and a vertical fracture of the posterior ilium that extends from the middle of the sacroiliac joint and exits the iliac crest superiorly. The posterior superior iliac spine remains firmly attached to the sacrum via the superior portion of the posterior ligamentous complex [4]. Day et al. proposed the presently accepted classification with three different fracture types and the resultant treatment recommendations were dependent on the amount of SI-joint involvement [9].

According to the Young-Burgess classification, the crescent fractures are part of LC 2 injuries and thus considered horizontally unstable but vertically stable [6, 43]. In contrast, recent reports have indicated that some crescent fractures can be vertically unstable. Zong et al. reported on four translationally unstable crescent fractures in a group of 31 patients [44]. Approximately 30–40% of all crescent fractures are classified as type-C injuries according to Tile [22, 35]. It should be considered that between 12 - 30% of crescent-type fractures do not fit into the Day classification scheme [7].

Overall, the 2018 AO/OTA pelvic ring fracture classification created a type-B injury problem (Table 3). The “new” mixing of injury mechanisms, instability patterns, and change of hierarchy made the classification of type-B injuries difficult. This clinical problem should be discussed in a wider forum. We recommend returning the classification to its previous form.

## Summary

The 1996 version of the AO/OTA pelvic ring fracture and dislocation classification seems to be optimal from the authors' perspective. In the 2018 AO/OTA classification, the main disadvantages are related to the reclassification of type-B pelvic ring injuries.

## Data Availability

No data was used for the research described in the article.
